# Data on four criteria for targeting the placement of conservation buffers in agricultural landscapes

**DOI:** 10.1016/j.dib.2016.04.006

**Published:** 2016-04-08

**Authors:** Zeyuan Qiu, Michael G. Dosskey, Yang Kang

**Affiliations:** a Department of Chemistry and Environmental Science, New Jersey Institute of Technology, Newark, NJ, United States; b USDA Forest Service, National Agroforestry Center, Lincoln, NE, United States; c Department of Statistics, Columbia University, New York, NY, United States

## Abstract

Four criteria are generally used to prioritize agricultural lands for placing conservation buffers. The criteria include soil erodibility, hydrological sensitivity, wildlife habitat, and impervious surface rate that capture conservation buffers’ benefits in reducing soil erosion, controlling runoff generation, enhancing wildlife habitat, and mitigating stormwater impacts, respectively. This article describes the data used to derive the values of those attributes and a scheme to classify the values in multi-criteria analysis of conservation buffer placement in “Choosing between alternative placement strategies for conservation buffers using borda count” [1].

## Specifications Table

**Table:** 

Subject area	*Ecology*
More specific subject area	*Conservation buffers*
Type of data	*Tables, figures*
How data was acquired	*Maps generated from readily available spatial data using ArcMap 10.3 developed by ESRI*
Data format	*Processed*
Experimental factors	*Primarily based on a 10-m digital elevation model and the 2002 land use/cover data developed and maintained by New Jersey Department of Environmental Protection (NJDEP) and a digital soil database maintained by the U.S. Department of Agriculture Natural Resources Conservation Service (NRCS)*
Experimental features	*Presented spatially in the original and classified values*
Data source location	*Raritan River Basin, Central New Jersey, USA*
Data accessibility	*Data are within this article*

## Value of the data

• Each of four criteria can be useful indicators for prioritizing conservation efforts in landscapes.
• A localized classification system is a powerful tool to balance the subjective preferences of stakeholders and the objective measurement of natural resource conditions in resource management decision-making.
• The method for analyzing the data and the classification system are useful tools for making critical decisions on conservation buffer placement.

## 1 Data

This study is to present the data on four criteria values and a scheme to classify those values for multi-criteria analysis of prioritizing agricultural lands for conservation buffer placement in Raritan River Basin in central New Jersey, USA [1], [2]. The data include soil erodibility, hydrological sensitivity, wildlife habitat, and impervious surface rate that capture buffers’ benefits in reducing soil erosion, controlling runoff generation, enhancing wildlife habitat, and mitigating stormwater impacts, respectively. The data were derived from readily available spatial data on resource conditions in landscapes.

## 2 Experimental design, materials and methods

### 2.1 Soil erodibility

Soil erodibility is approximated by soil erodibility index (SEI) [3]. To derive SEI, the value of rainfall and runoff intensity was estimated from the annualized isoerodent map for the eastern United States and was set at 160 for the region [4]. The slope length factor and slope steepness factor were derived from a 10-meter digital elevation model (DEM) maintained by NJDEP. The susceptibility of soil to water erosion and the soil loss tolerance factor were extracted from the Soil Survey Geographic database (SSURGO) maintained by NRCS. The estimated SEI ranged from zero to 14,228 and its distribution in the basin is shown in Supplementary Fig. 1a. The index was classified into five classes based on the following classification scheme (Table 1) and the spatial distribution of the five soil erodibility classes is presented in Supplementary Fig. 1b.

### 2.2 Hydrological sensitivity

Hydrological sensitivity is approximated by a modified topographic index based on the VSA hydrology [5]. Similarly, the topographic index was derived from the NJDEP DEM and NRCS SSURGO for the basin. The topographic index values for the 10-m grids in the basin ranged from 0.13 to 30.37. The spatial distribution of the index in the basin was presented in Supplementary Fig. 2a. The basin was further divided into 10 hydrological zones of equal size based on the topographic index values with Zone 10 being the most hydrologically sensitive and Zone 1 the least sensitive. The spatial distribution of ten hydrological zones is presented in Supplementary Fig. 2b.

### 2.3 Wildlife habitat

Wildlife habitat condition in the basin was evaluated using the results from the NJDEP Nongame and Endangered Species Program׳s Landscape Project Version 2.1, which identified five general habitat types for rare species including forest, forested wetland, grassland, emergent wetland, and beach, as well as three specific habitat areas: bald eagle foraging areas; urban peregrine falcon nests; and wood turtle habitat. The wildlife habitat value is the sum of the habitat rank for potential presence of species of concern (rank 1–5) and the presence of bald eagle, falcon or wood turtle in forested wetlands and emergent wetlands (1). The final wildlife habitat values ranged from zero to eight and their spatial distribution is presented in Supplementary Fig. 3a. The wildlife habitat was further divided into six classes based on the classification scheme presented in Table 2, whose spatial distribution in the basin is presented in Supplementary Fig. 3b.

### 2.4 Impervious surface

Impervious surfaces are built-up surfaces (i.e., rooftops, sidewalks, roads, and parking lots) covered by impenetrable materials, such as asphalt, concrete, brick, and stone. Impervious surface rate was extracted from the NJDEP 2002 land use/cover data for the basin. The land use/cover data estimate the impervious surface rate for each land use/cover polygon using aerial photography and reported it in 5% increments between 0% and 100%. The spatial distribution of the impervious surface rate in the basin is presented in Supplementary Fig. 4a. The basin was further divided into five different impervious surface classes following the classification scheme in Table 3. The spatial distribution of the five impervious surface classes is presented in Supplementary Fig. 4b.

## Figures and Tables

**Table 1:** A classification scheme for soil erodibility.

Soil erodibility class score	Description	Soil erodibility index
1	Non-erodible	≤2
2	Low erodibility	2–5
3	Medium erodibility	5–8
4	High erodibility	8–12
5	Extremely high erodibility	>12

**Table 2:** A classification scheme for wildlife habitat.

Wildlife habitat class score	Description	Wildlife habitat value
0	No	0
1	Poor	1
2	Fair	2
3	Good	3
4	Excellent	4
5	Outstanding	5–8

**Table 3:** A classification scheme for impervious surface.

Impervious surface class score	Description	Impervious surface rate (%)
1	Low impact	0
3	Medium impact	5
5	High impact	10
7	Very high impact	15
9	Excessively high impact	≥20

## Footnotes

Appendix A Supplementary data associated with this article can be found in the online version at doi:10.1016/j.dib.2016.04.006.
