# Supplementary figures and images for: Data on four criteria for targeting the placement of conservation buffers in agricultural landscapes
Source: Data Brief. 2016 Apr 8;7:1254–7. doi: 10.1016/j.dib.2016.04.006 (PMC4865647; doi:10.1016/j.dib.2016.04.006)

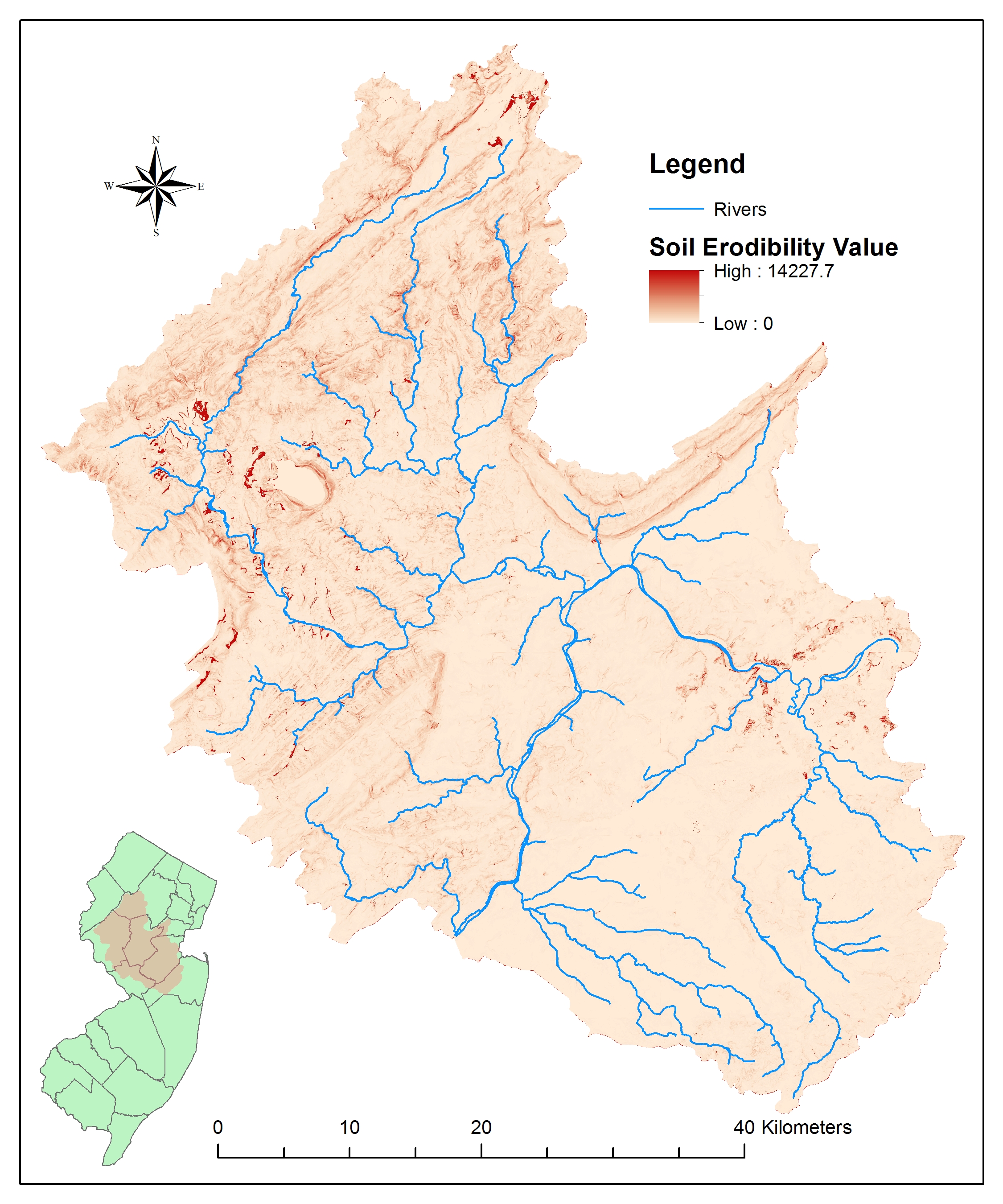

Supplement: Supplementary file 1 — Supplementary material [file mmc1.zip › Figure1a.jpg]

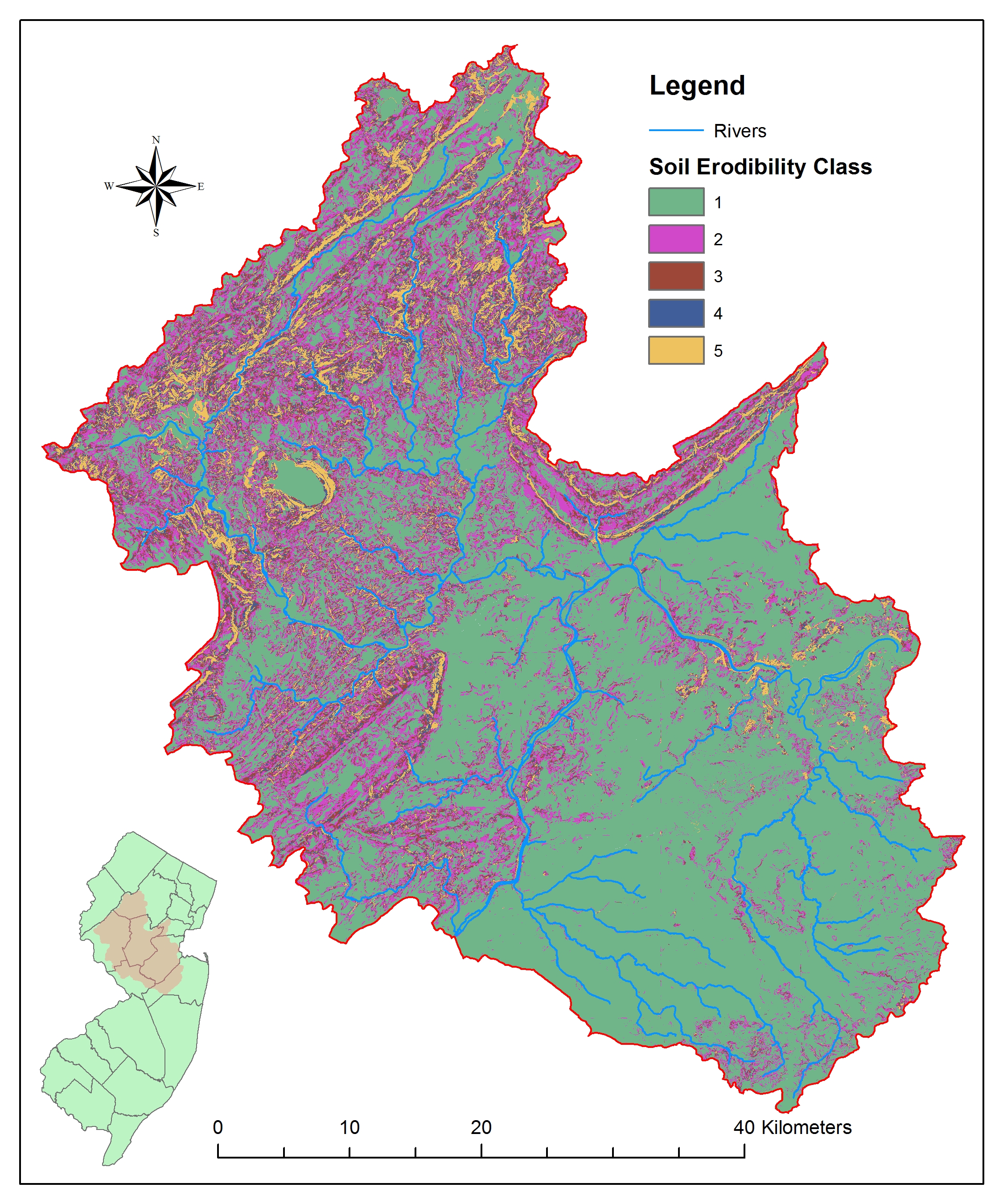

Supplement: Supplementary file 1 — Supplementary material [file mmc1.zip › Figure1b.jpg]

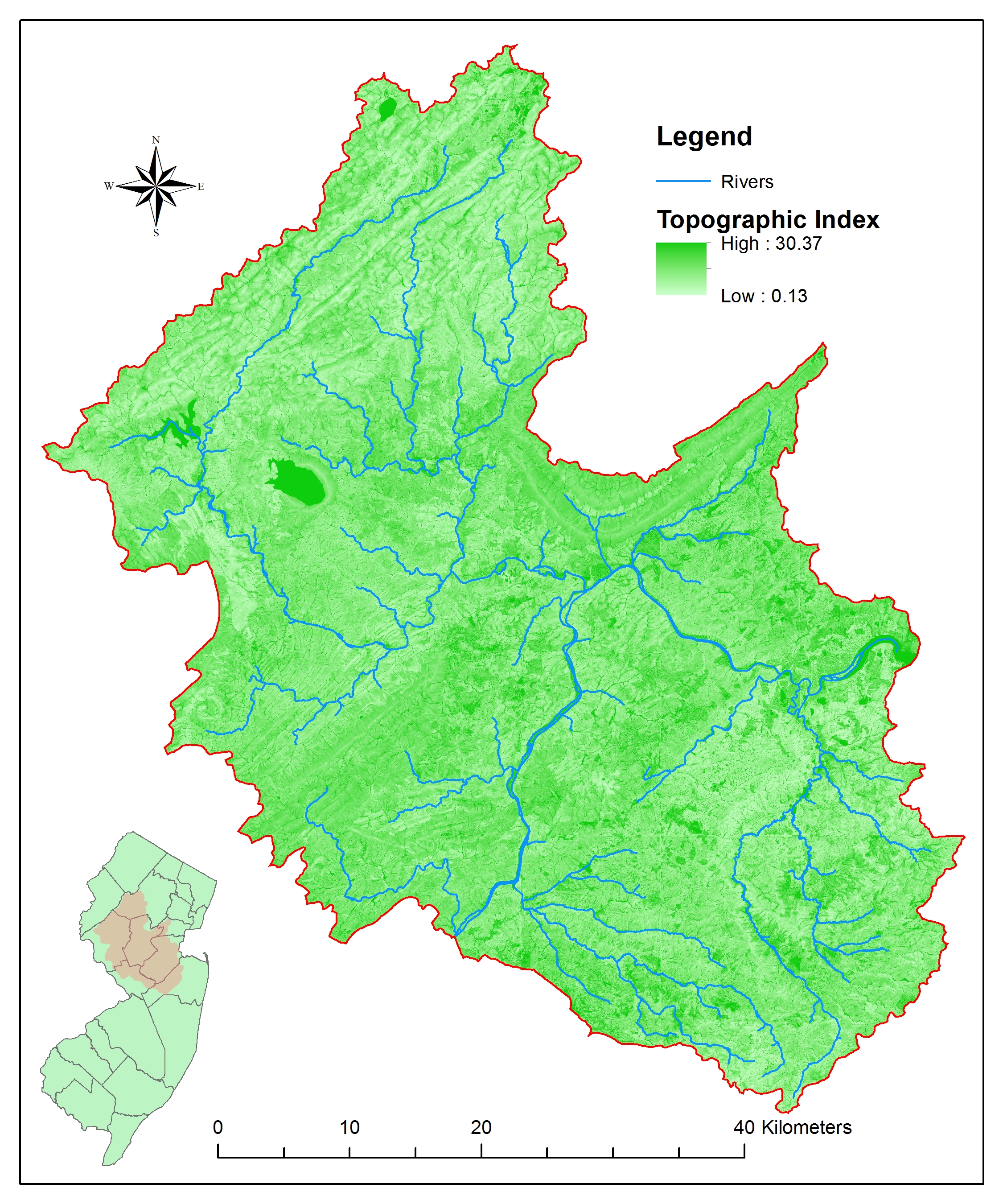

Supplement: Supplementary file 1 — Supplementary material [file mmc1.zip › Figure2a.jpg]

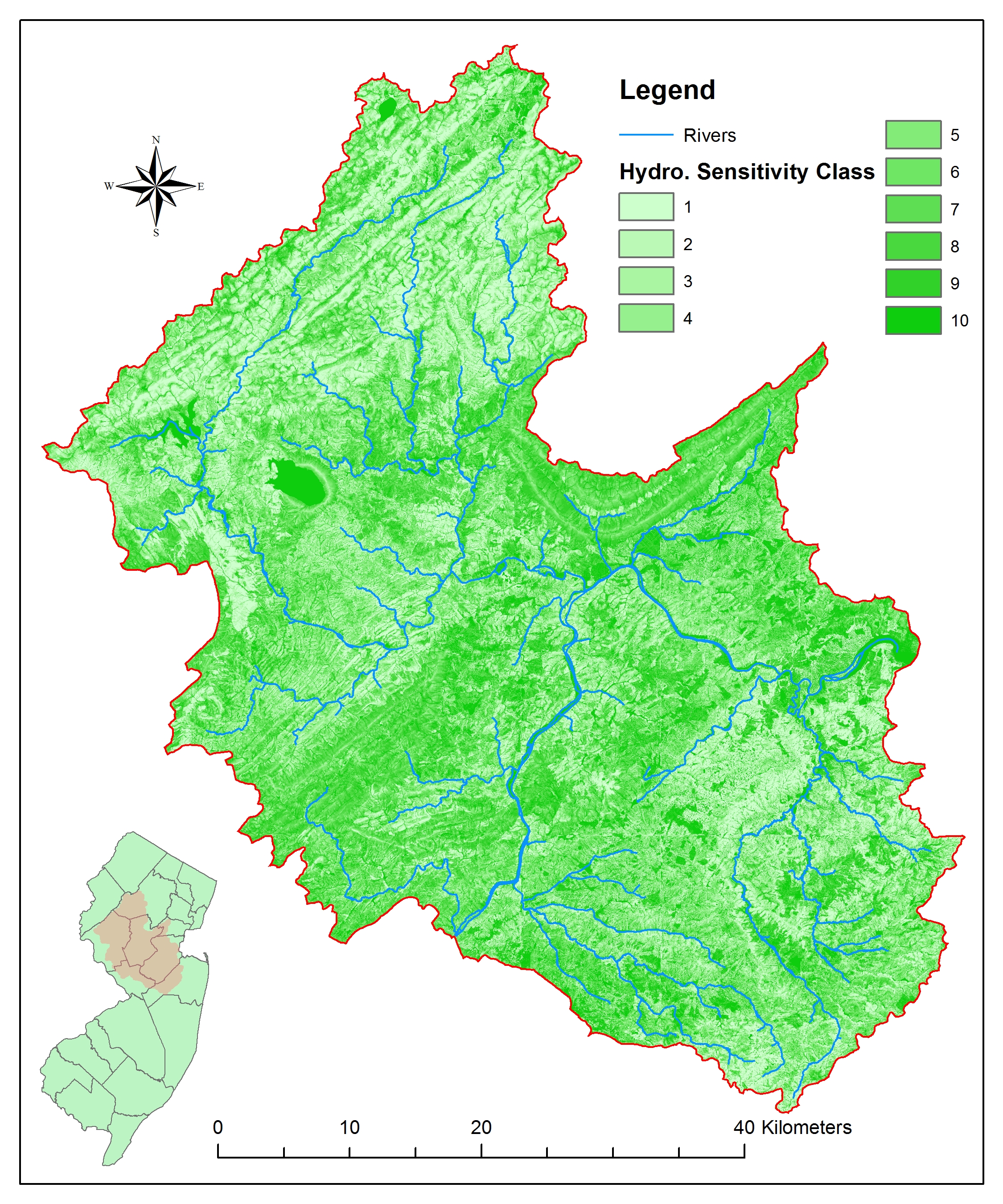

Supplement: Supplementary file 1 — Supplementary material [file mmc1.zip › Figure2b.jpg]

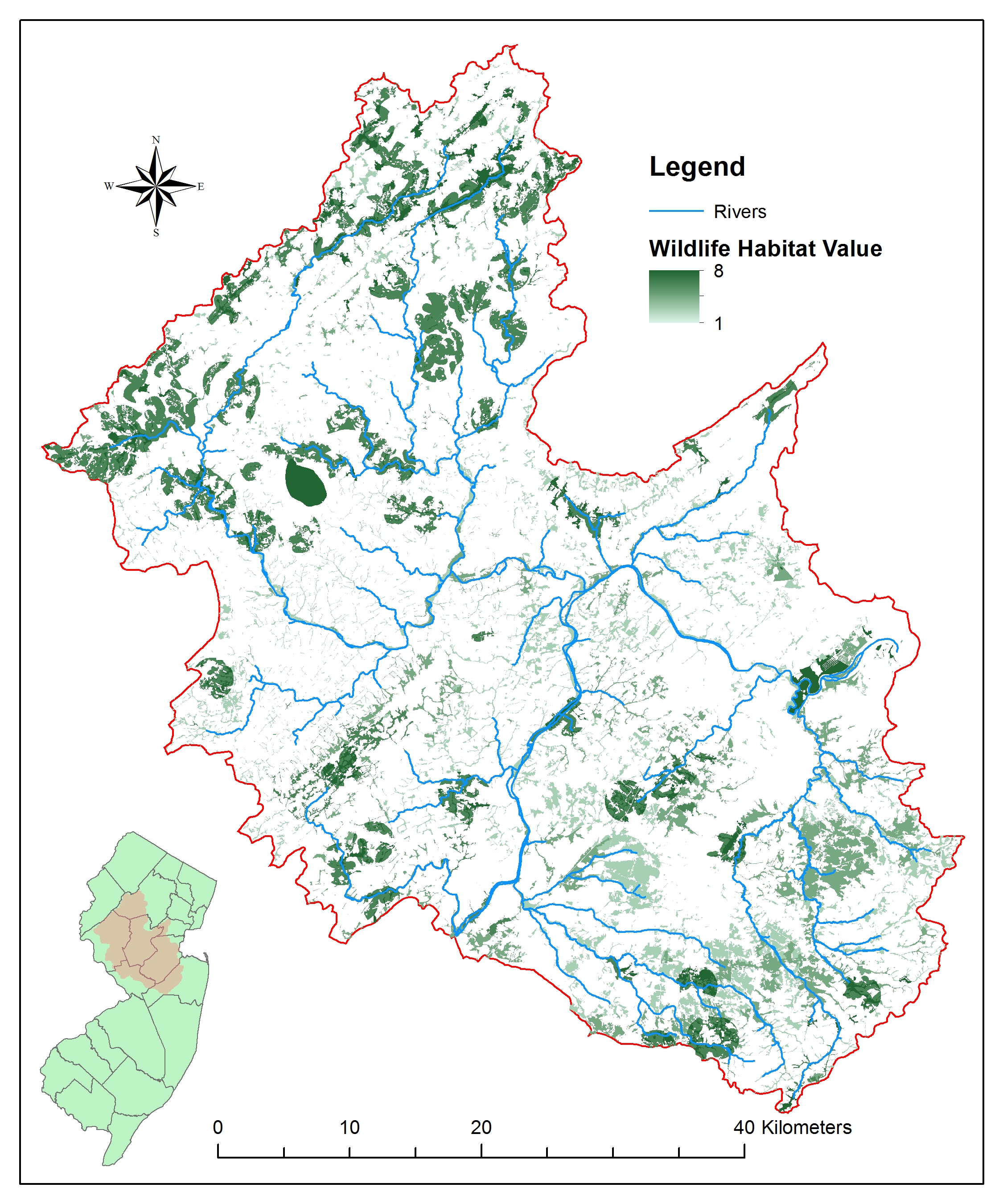

Supplement: Supplementary file 1 — Supplementary material [file mmc1.zip › Figure3a.jpg]

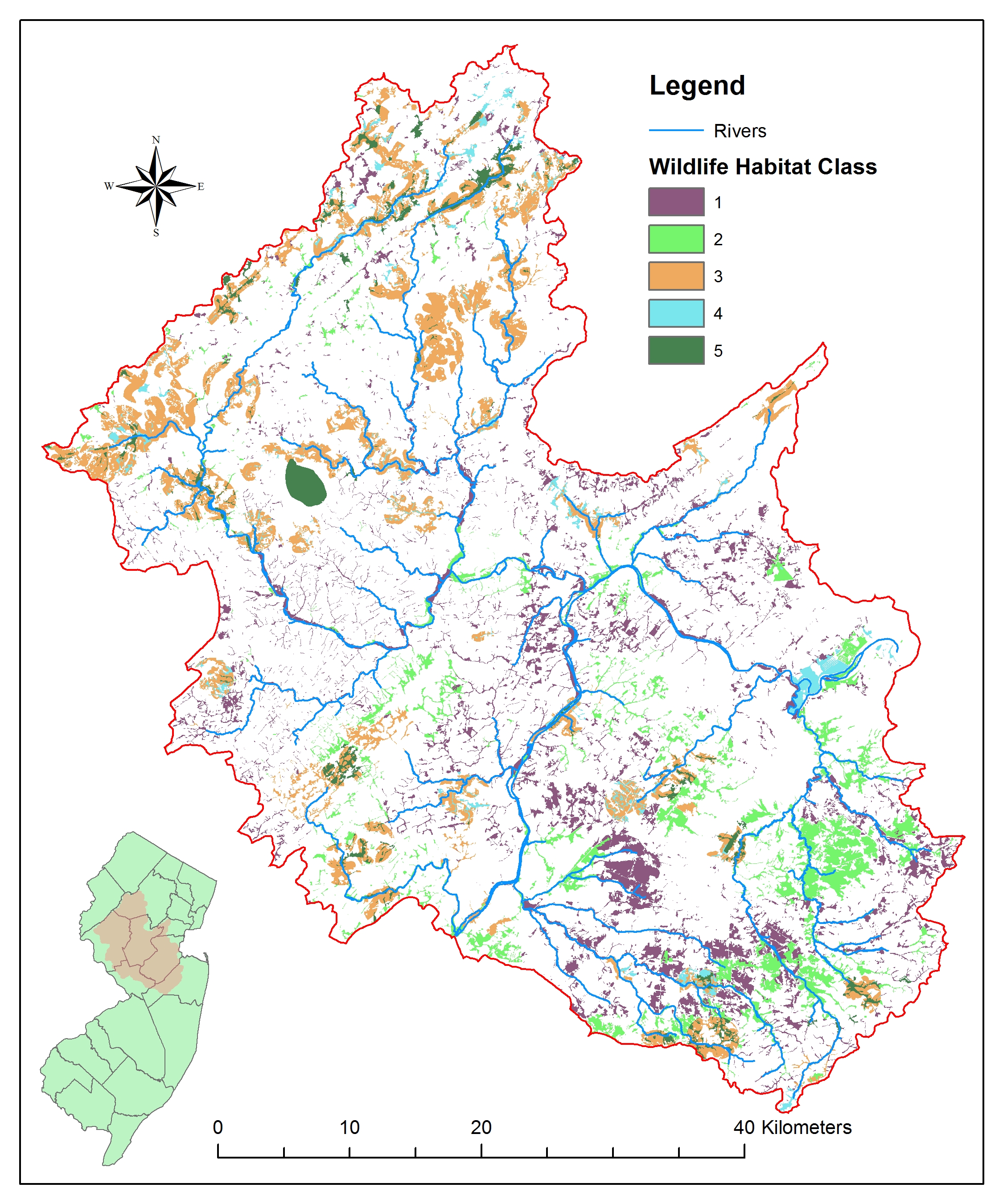

Supplement: Supplementary file 1 — Supplementary material [file mmc1.zip › Figure3b.jpg]

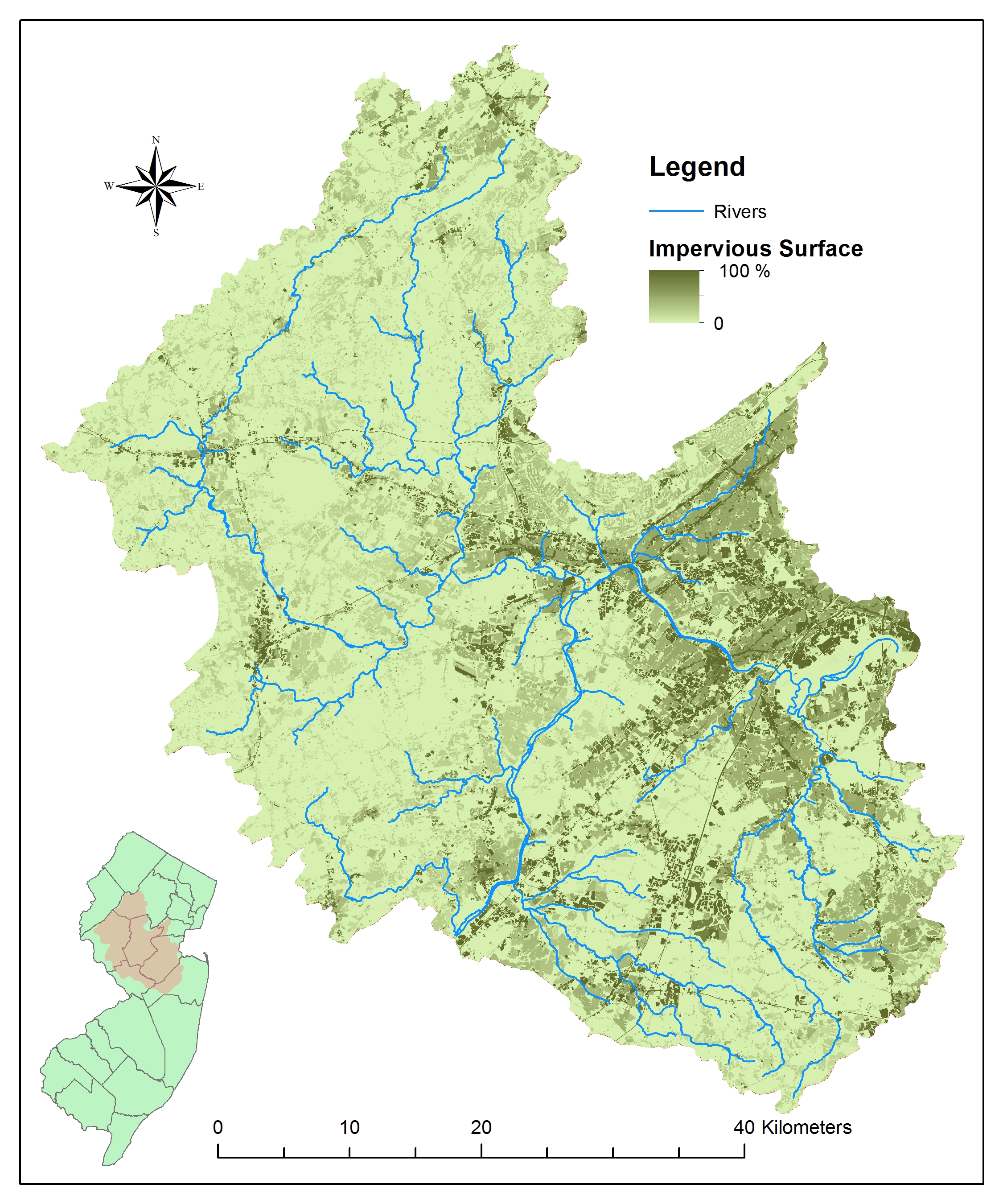

Supplement: Supplementary file 1 — Supplementary material [file mmc1.zip › Figure4a.jpg]

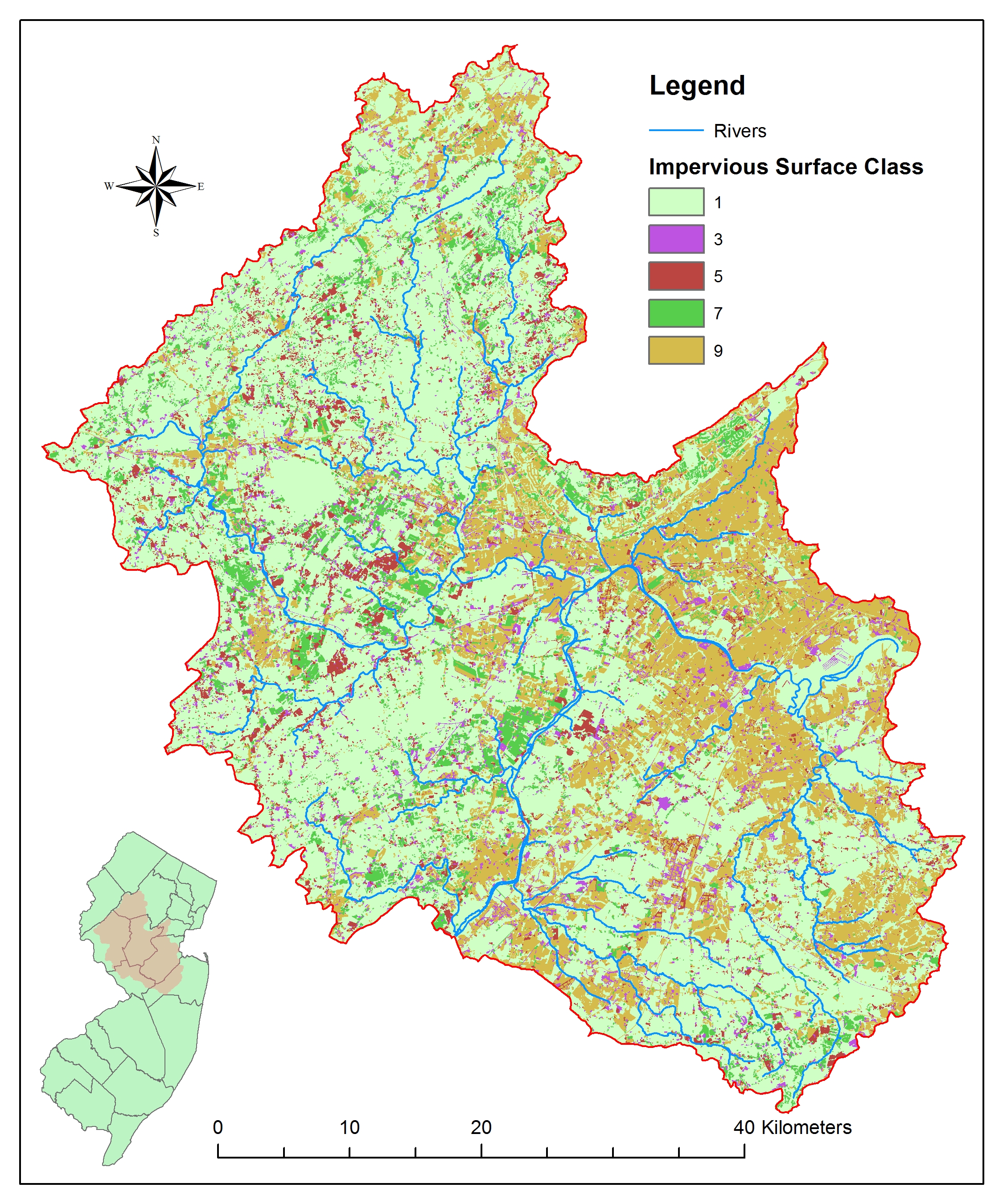

Supplement: Supplementary file 1 — Supplementary material [file mmc1.zip › Figure4b.jpg]
