# Supplementary material for: Data on four criteria for targeting the placement of conservation buffers in agricultural landscapes
Source: Data Brief. 2016 Apr 8;7:1254–7. doi: 10.1016/j.dib.2016.04.006 (PMC4865647; doi:10.1016/j.dib.2016.04.006)
Supplement: Supplementary file 2 — Supplementary material [file mmc2.pdf]

Data on Four Criteria for Targeting the Placement of Conservation Buffers in Agricultural Landscapes (Manuscript DIB-D-16-00223)

Data in Brief

Conflict of Interest Declaration

We wish to confirm that there are no known conflicts of interest associated with this publication and there has been no significant financial support for this work that could have influenced its outcome.

We confirm that the manuscript has been read and approved by all named authors and that there are no other persons who satisfied the criteria for authorship but are not listed. We further confirm that the order of authors listed in the manuscript has been approved by all of us.

We confirm that we have given due consideration to the protection of intellectual property associated with this work and that there are no impediments to publication, including the timing of publication, with respect to intellectual property. In so doing we confirm that we have followed the regulations of our institutions concerning intellectual property.

We understand that the Corresponding Author is the sole contact for the Editorial process (including Editorial Manager and direct communications with the office). He/she is responsible for communicating with the other authors about progress, submissions of revisions and final approval of proofs. We confirm that we have provided a current, correct email address which is accessible by the Corresponding Author and which has been configured to accept email from

Zeyuan Qiu: zeyuan.qiu@njit.edu
Michael G. Dosskey: mdosskey@fs.fed.us
Yang Kang: yangkang@stat.columbia.edu

Signed by all authors as follows:

[illegible]
Zeyuan Qiu

3/29/2016
Date

M.G. Dosskey
Michael G. Dosskey

3-29-16
Date

[illegible]
Yang Kang

03/29/2016
Date
